# Supplementary material for: Effects of Reablement on the Independence of Community-Dwelling Older Adults with Mild Disability: A Randomized Controlled Trial
Source: Int J Environ Res Public Health. 2019 Oct 17;16(20):3954. doi: 10.3390/ijerph16203954 (PMC6843923; doi:10.3390/ijerph16203954)
Supplement: Supplementary file 1 [file ijerph-16-03954-s001.zip › ijerph-602679-supple. proofed/ijerph-602679-Table and Figure.pdf]

International Journal of
Environmental Research and Public Health

MDPI

Supplementary Materials:

Table S1. Options and their corresponding codes used in the assessment of IADL and ADL.

Item	Options and Their Corresponding Codes
IADL	
Getting around outdoors	0 = at least once a week; 1 = at least once a month/less than once a month
Taking medication	0 = independent, 1 = need help/dependent
Managing money	0 = independent, 1 = need help/dependent
Shopping for groceries	0 = independent, 1 = need supervision/need help/dependent
Preparing food	0 = independent, 1 = need supervision/need help/dependent
ADL	
Ability to turn over in bed	0 = independent/need equipment; 1 = dependent
Sitting up in bed	0 = independent/need equipment; 1 = dependent
Sitting for 10 minutes	0 = independent/need upper limb; 1 = need help/dependent
Standing on both feet for 10 seconds	0 = independent/need equipment; 1 = dependent
Walking 5 meters	0 = independent/need equipment; 1 = dependent
Standing up	0 = independent/need equipment; 1 = dependent
Standing on one foot for 1 second	0 = independent/need equipment; 1 = dependent
Washing body	0 = independent; 1 = need help/dependent/does not do
Caring for nails	0 = independent; 1 = need help/dependent
Ability to see	0 = no problem; 1 = can read the eye-chart from 1-meter distance/can read the eye-chart in front of one's eyes/with vision problems/unable to assess the ability
Ability to hear	0 = no problem; 1 = mild difficulty/moderate difficulty/severe difficulty/unable to assess the ability
Moving from bed to wheelchair, wheelchair to toilet, and so on	0 = independent, 1 = need supervision/need help/dependent
Moving from room to room	0 = independent, 1 = need supervision/need help/dependent
Swallowing	0 = independent, 1 = need supervision/difficulty
Eating	0 = independent, 1 = need supervision/need help/dependent
Urinating	0 = independent, 1 = need supervision/need help/dependent
Defecating	0 = independent, 1 = need supervision/need help/dependent
Oral care	0 = independent, 1 = need help/dependent
Washing face	0 = independent, 1 = need help/dependent
Styling hair	0 = independent, 1 = need help/dependent
Putting on and/or taking off upper wear	0 = independent, 1 = need supervision/need help/dependent
Putting on and/or taking off lower wear	0 = independent, 1 = need supervision/need help/dependent

ADL, activities of daily living; IADL, instrumental activities of daily living.

Table S2. Use of long-term care services by different population sets during the 5-month intervention period.

Population	Intervention Group	Control Group
ITT population		
Home-visit services, average visits	15.6 (20.6)	16.3 (32.5)
Commuting services, average visits	26.1 (18.7)	19.9 (15.9)
Short-stay services, average days	0.1 (1.1)	0.1 (1.1)
Facility services, average days	0.3 (3.8)	0.0 (0.0)
Medical care management counseling, average visits	0.5 (3.6)	0.3 (2.7)
Rental of assistive equipment, average days	50.6 (69.8)	54.6 (70.6)
FAS population		
Home-visit services, average visits	16.1 (19.9)	17.5 (33.3)
Commuting services, average visits	29.8 (17.8)	21.2 (15.6)
Short-stay services, average days	0.1 (1.2)	0.1 (1.1)
Facility services, average days	0.0 (0.0)	0.0 (0.0)
Medical care management counseling, average visits	0.3 (3.6)	0.3 (2.8)
Rental of assistive equipment, average days	53.0 (70.7)	58.3 (71.5)
PPS population		
Home-visit services, average visits	16.4 (20.3)	17.7 (33.7)
Commuting services, average visits	31.1 (17.4)	21.5 (15.7)
Short-stay services, average days	0.1 (1.3)	0.2 (1.1)
Facility services, average days	0.0 (0.0)	0.0 (0.0)
Medical care management counseling, average visits	0.3 (3.8)	0.4 (2.9)
Rental of assistive equipment, average days	53.0 (70.8)	59.9 (71.9)

FAS, full analysis set; ITT, intention-to-treat; PPS, per-protocol set; SD, standard deviation. The values inside parentheses denote the SD values for the two groups in each row. The FAS population refers to participants who have received the allocated interventions at least once, and the PPS population refers to those who have received more than half of the allocated interventions.

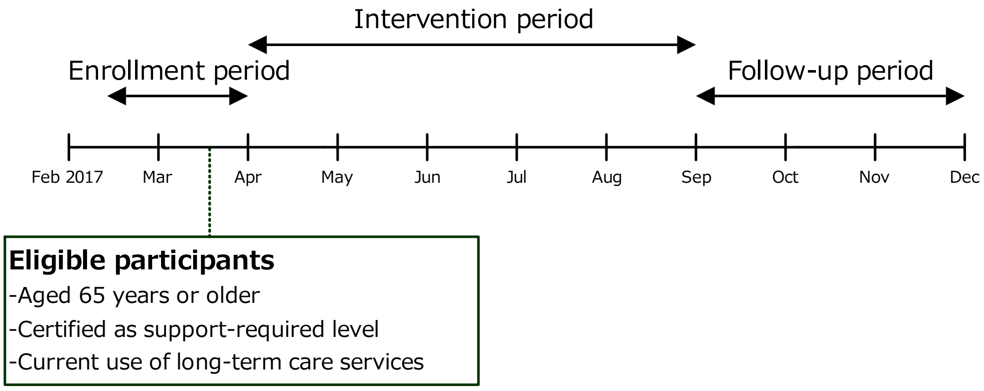

Figure S1. Timeline of the trial.

Subgroup	No./Total No. (%) Intervention	Control	Difference, % (95% CI)	P value
Total	17/144 (11.8)	1/173 (0.6)	11.2 (5.8, 16.6)	0.000
Age, y				
65−74	1/25 (4.0)	0/32 (0.0)	4.0 (−3.7, 11.7)	0.342
75−84	11/90 (12.2)	1/101 (1.0)	11.2 (4.2, 18.3)	
≥85	5/29 (17.2)	0/40 (0.0)	17.2 (3.5, 31.0)	
Long−term care service				
Prevalent user	16/133 (12.0)	0/155 (0.0)	12.0 (6.5, 17.6)	0.985
New user	1/11 (9.1)	1/18 (5.6)	3.5 (−16.5, 23.6)	
Support required level				
1	10/80 (12.5)	1/94 (1.1)	11.4 (3.9, 19.0)	0.794
2	7/64 (10.9)	0/79 (0.0)	10.9 (3.3, 18.6)	
Sex				
Female	12/100 (12.0)	1/110 (0.9)	11.1 (4.5, 17.7)	0.979
Male	5/44 (11.4)	0/63 (0.0)	11.4 (2.0, 20.7)	
Dementia				
Without	10/88 (11.4)	1/102 (1.0)	10.4 (3.5, 17.3)	0.808
With	7/56 (12.5)	0/71 (0.0)	12.5 (3.8, 21.2)	
Number of impaired ADL				
0	2/32 (6.2)	1/42 (2.4)	3.9 (−5.7, 13.4)	0.315
1	8/45 (17.8)	0/57 (0.0)	17.8 (6.6, 28.9)	
≥2	7/67 (10.4)	0/74 (0.0)	10.4 (3.1, 17.8)	
Number of impaired IADL				
0	4/48 (8.3)	0/44 (0.0)	8.3 (0.5, 16.2)	0.532
1	3/31 (9.7)	0/41 (0.0)	9.7 (−0.7, 20.1)	
≥2	10/65 (15.4)	1/88 (1.1)	14.2 (5.2, 23.3)	

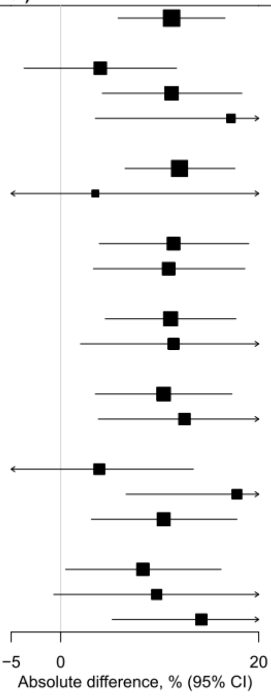

Figure S2. Effects of reablement on the independence from long-term care services of post-hoc subgroups in the full set analysis population, which excluded participants who had not received the allocated interventions. The *p*-values for subgroup comparisons correspond to the test for interaction. ADL, activities of daily living; CI, confidence interval; IADL, instrumental activities of daily living; y, years.

Subgroup	No./Total No. (%) Intervention	Control	Difference, % (95% CI)	P value
Total	14/125 (11.2)	1/168 (0.6)	10.6 (5.0, 16.3)	0.000
Age, y				
65−74	1/23 (4.3)	0/32 (0.0)	4.3 (−4.0, 12.7)	0.539
75−84	9/74 (12.2)	1/96 (1.0)	11.1 (3.4, 18.8)	
≥85	4/28 (14.3)	0/40 (0.0)	14.3 (1.3, 27.2)	
Long−term care service				
Prevalent user	13/116 (11.2)	0/151 (0.0)	11.2 (5.5, 16.9)	0.753
New user	1/9 (11.1)	1/17 (5.9)	5.2 (−18.2, 28.6)	
Support required level				
1	8/66 (12.1)	1/93 (1.1)	11.0 (2.9, 19.2)	0.746
2	6/59 (10.2)	0/75 (0.0)	10.2 (2.5, 17.9)	
Sex				
Female	9/83 (10.8)	1/108 (0.9)	9.9 (3.0, 16.8)	0.808
Male	5/42 (11.9)	0/60 (0.0)	11.9 (2.1, 21.7)	
Dementia				
Without	9/78 (11.5)	1/99 (1.0)	10.5 (3.2, 17.9)	0.923
With	5/47 (10.6)	0/69 (0.0)	10.6 (1.8, 19.5)	
Number of impaired ADL				
0	2/26 (7.7)	1/42 (2.4)	5.3 (−5.9, 16.5)	0.322
1	7/39 (17.9)	0/55 (0.0)	17.9 (5.9, 30.0)	
≥2	5/60 (8.3)	0/71 (0.0)	8.3 (1.3, 15.3)	
Number of impaired IADL				
0	4/42 (9.5)	0/42 (0.0)	9.5 (0.6, 18.4)	0.798
1	2/24 (8.3)	0/41 (0.0)	8.3 (−2.7, 19.4)	
≥2	8/59 (13.6)	1/85 (1.2)	12.4 (3.4, 21.4)	

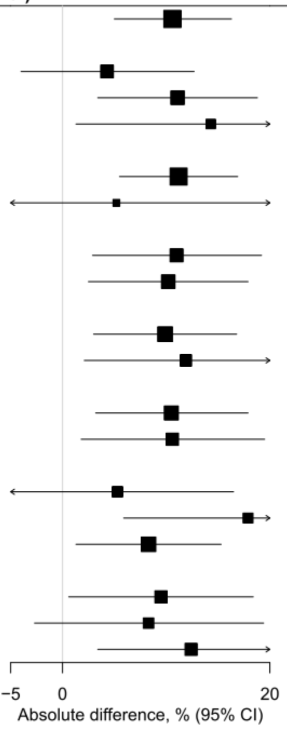

Figure S3. Effects of reablement on the independence from long-term care services of post-hoc subgroups in the per-protocol set population, which excluded participants who had not received more than half of the allocated interventions. The *p*-values for subgroup comparisons correspond to the test for interaction. ADL, activities of daily living; CI, confidence interval; IADL, instrumental activities of daily living; y, years.
