# Supplementary material for: Effects of Reablement on the Independence of Community-Dwelling Older Adults with Mild Disability: A Randomized Controlled Trial
Source: Int J Environ Res Public Health. 2019 Oct 17;16(20):3954. doi: 10.3390/ijerph16203954 (PMC6843923; doi:10.3390/ijerph16203954)
Supplement: Supplementary file 1 [file ijerph-16-03954-s001.zip › ijerph-602679-supple. proofed/ijerph-602679-supplementary File 1.pdf]

Assessment sheet for self-management

No /

Name	Care goal setting	Process to achieve the goal

Date	Service	Condition	Daily activities to achieve the goal					
			1	2	3	4	Good point/bad point	Diary / impression
			Stand up from a chair (physical activities)	Swallowing exercises (oral health)	Water intake (nutrition)	xxxx		
Frequency ->			5 times	5 times	1000 ml/day	xxxx		
1-Apr	Commuting service	normal	4	5	1000	xxxx	Generally good	I made craftwork.
2-Apr	CoMMIT	good	2	5	900	xxxx	Should improve activity 1	I learned new self-training.
3-Apr		good	0	0	1200	xxxx	Should improve activity 1 and 2	I watered the flowers.
4-Apr		good	4	5	900	xxxx	Generally good	I chatted with neighbors.
5-Apr	Home-visit service	normal	0	5	900	xxxx	Should improve activity 1	
6-Apr		normal	0	5	900	xxxx	Should improve activity 1	I went to my grandson's house.
7-Apr		normal	5	5	1000	xxxx	Should improve activity 1	I went to shopping on a bicycle.

Comment from family	Comment from participant		Advise from provider
