# Supplementary material for: Effects of Reablement on the Independence of Community-Dwelling Older Adults with Mild Disability: A Randomized Controlled Trial
Source: Int J Environ Res Public Health. 2019 Oct 17;16(20):3954. doi: 10.3390/ijerph16203954 (PMC6843923; doi:10.3390/ijerph16203954)
Supplement: Supplementary file 1 [file ijerph-16-03954-s001.zip › ijerph-602679-supple. proofed/ijerph-602679-Supplementary File2.pdf]

Supplementary File 2

寝屋川市
NEYAGAWA CITY

介護予防手帳
短期集中モデル版

A booklet for preventing long-term care needs

名前 ________________________

Supplementary File 2

この手帳の活用について

How to use this booklet

　この手帳は、寝屋川市の短期集中通所型サービスを利用したみなさんが、引き続き目標をもって、 地域で自分らしく暮らしつづけることができるように作成しました。

　年齢を重ねるごとに筋力が落ちたり、生活環境が変わったりして、これまでどおりの生活ができなくなることもあるかもしれませんが、いつまでも自分でできることは自分で行い、好きなことを楽しみながら暮らすことが大事だと考えています。

　そのためには、定期的に体を動かすこと、人と交流し、たくさん笑うことなど、心身ともに健康に過ごすために効果的な取り組みを継続していただくことが重要です。

　この手帳は、日々の生活をふりかえり、新たに始めてみたい活動を考えたり、気力、体力の低下を感じたときの相談先を確認していただくなど、みなさんがいつまでも自分らしく充実した生活を送るために活用していただきたいと考えています。

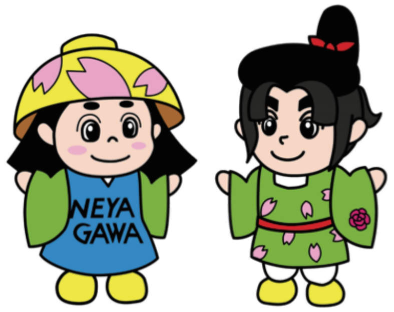

自分らしい生活を続けるためのチェック項目

Assessment for preventing long-term care needs

1) 社会参加のこと

Social participation

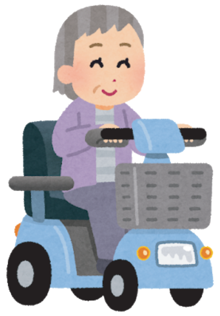

- ☐ 役割(趣味、仕事)を続けている
- ☐ いままで通り外出している（外出先）
- ☐ いままで通り外出している（頻度）
- ☐ いままで通り友人・知人と会っている
- ☐ 誰かの役に立っていると思う

2) お口のこと

Oral health

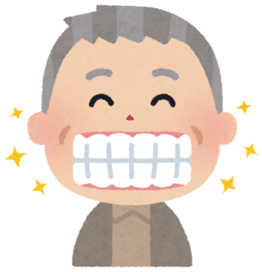

- ☐ おいしく食べられている
- ☐ スムーズに飲めている
- ☐ 楽しくおしゃべりできている
- ☐ たくさん笑っている
- ☐ 歯科医院に定期的に通っている

3) 食べること

Nutrition

- ☐ 1日3回食事をしている
- ☐ 3回の食事に1回は肉・魚・卵・豆腐類が必ず入っている
- ☐ 味付けはうす味にしている
- ☐ 水分を食事以外で1日1,000cc以上飲んでいる
- ☐ 便は毎日出ている

4) 運動のこと

Physical activities

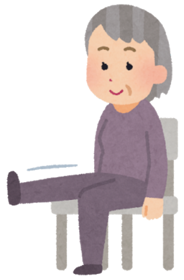

- ☐ 自主トレとして短期事業所で行った
  ①柔軟②筋力③バランスのトレーニングを
  1日1回行っている

わたしの「これまで・今・これから」

Activities in the past, current, and future

Activities that I want to do in the future.

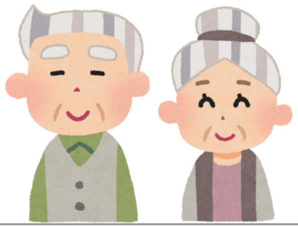

これから

これまで・今

Activities that I had been able to do in the past

Activities that I can regain in the previous 3 months.

- ○ これからしたいこと、つながりたいところやつながりたい人など
- ○ この3ヶ月で出来るようになったこと、今していることなど
- ○ これまで出来ていたこと、つながっていたところやつながっている人など

さんへの寄せ書き

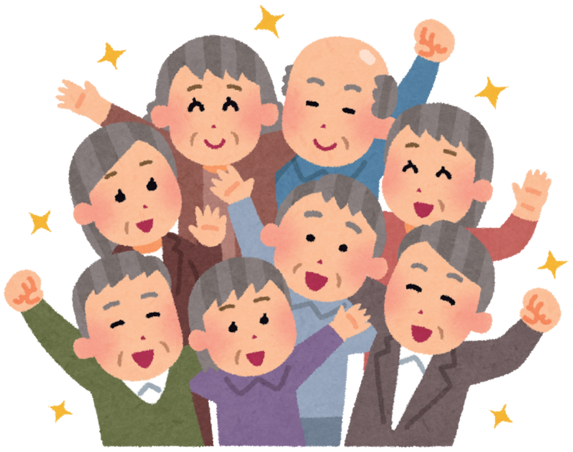

こういうことがあればすぐ相談してください

Situations that I should seek help as soon as possible.

- ☐
- ☐
- ☐
- ☐
- ☐

3ヶ月を振り返って・これからへの一言

Any comments

相談するところ

Resources for consultation.

- ● 電話：
- ● 電話：
- ● 電話：

寝屋川市 福祉部 高齢介護室
〒572-8533 大阪府寝屋川市池田西町28番22号
電話：072-838-0372（代表） 発行：2018年8月
